# A Comparison of the Clinicoradiological Outcomes of Intertrochanteric Fractures Treated Using Proximal Femoral Nail and Proximal Femoral Nail Anti-rotation

**DOI:** 10.7759/cureus.60639

**Published:** 2024-05-19

**Authors:** Shubham Bhardwaj, Harshal Sakale, Alok C Agrawal, Bikram Kar, Rudra Narayan Dash, Alok Rai, Ankit Kumar Garg

**Affiliations:** 1 Orthopedics, All India Institute of Medical Sciences, Raipur, IND; 2 Orthopedic Surgery, All India Institute of Medical Sciences, Raipur, IND

**Keywords:** proximal femoral nail anti-rotation, proximal femoral nail, cleveland index, femoral neck-shaft angle, modified harris hip score, femur intertrochanteric fracture

## Abstract

Background

Managing intertrochanteric fractures presents challenges for orthopedic surgeons, not only in fixing the fracture but also in preventing and managing associated complications, especially in the vulnerable geriatric population. Cephalomedullary nails are commonly used for surgical fixation due to their favorable functional profile, which preserves the hip's abductor lever arm and proximal femur anatomy. However, there's a lack of data comparing two major options: proximal femoral nail (PFN) and proximal femoral nail anti-rotation (PFNA). This study aimed to compare the radiological fracture reduction and fixation as well as functional outcomes of these two implants in treating intertrochanteric fractures.

Methods

The study, spanning 24 months, involved a prospective comparative design. Participants included patients diagnosed with intertrochanteric femur fractures classified as AO Type 31 A1, AO Type 31 A2, and AO Type 31 A3. Fifty patients were evenly distributed into PFN and PFNA groups. Preoperatively, clinical and radiological assessments were conducted, along with serum vitamin D level measurements. Surgeries, performed under anesthesia with image intensifier guidance, followed defined reduction and implant insertion protocols for each group. Postoperatively, evaluations were conducted up to six months, examining parameters such as tip-apex distance (TAD), Cleveland index, and modified Harris hip score, while documenting intraoperative duration and blood loss. Data analysis utilized the statistical software Statistical Package for Social Sciences (SPSS), version 22.0 (IBM Corp., Armonk, NY), employing descriptive statistics, chi-square tests, independent t-tests, and paired t-tests, with significance set at p < 0.05.

Results

In our study, 50 patients were enrolled, with equal gender distribution (64.0% male, 36.0% female, p=1.000). The mean ages in the PFN and PFNA groups were 66.2 ± 9.8 years and 66.4 ± 11.3 years, respectively (p=0.936). All fractures united by six months, with no implant-related complications reported. PFNA showed significantly lower blood loss and shorter surgery durations (p<0.001). TAD and neck shaft angle were similar between groups (p=0.826, p=0.555). Cleveland index placement and modified Harris hip score improvement were comparable (p=0.836, p<0.001). Predominant vitamin D deficiency was observed in both groups.

Conclusion

PFNA offers measurable intraoperative benefits over conventional PFN in terms of operative time, blood loss, and need for fluoroscopic imaging. However, no statistically observable benefits were noted in postoperative functional outcomes or complications between the two implants.

## Introduction

Improved healthcare facilities have significantly increased life expectancy in developing nations like India, with expectations of further improvements in the future. Consequently, there is a growing need to focus healthcare research on the health concerns of the elderly population. Among these concerns, orthopedic issues, such as intertrochanteric fractures, are particularly relevant due to their impact on mobility and overall health in older individuals [1].

Intertrochanteric fractures, which involve the upper end of the femur between both trochanters, are closely associated with advancing age, with a male-to-female ratio of 1.5:1. Global incidence rates vary based on regional demographics but are projected to surpass 7 million annually within the next few decades [2]. This surge poses a substantial burden on healthcare systems, necessitating effective treatment strategies by orthopedic surgeons.

Traditionally, non-operative approaches for these fractures have been overshadowed by surgical methods, with most patients undergoing surgical fixation. Non-operative methods are typically reserved for patients deemed unsuitable for surgery due to comorbidities or pre-existing non-ambulatory conditions [3].

Surgical fixation commonly involves the use of implants such as dynamic hip screws or intramedullary devices like the proximal femoral nail (PFN). While dynamic hip screws suffice for stable fractures, they may inadequately stabilize unstable ones [3]. The PFN, although widely used for unstable fractures, is associated with complications such as screw cut out, back out, varus collapse, and rotational instability, particularly in elderly populations, necessitating revision surgery [4,5].

In response to these challenges, the proximal femoral nail anti-rotation (PFNA) was developed to enhance femoral head and neck stabilization using a single helical blade. Biomechanical studies suggest greater resistance to rotation and varus collapse due to enhanced bone compaction around the helical blade. Additionally, PFNA theoretically offers simplified surgical procedures, reduced operative times, and blood loss [6]. However, the translation of these theoretical benefits into real-world functional improvements requires further investigation.

To address this gap, we conducted a prospective study to compare the radiological and functional outcomes of intertrochanteric fractures treated with PFN and PFNA implants.

## Materials and methods

Study design and participants

The study was a 24-month-long prospective comparative study, initiated after obtaining approval (AIIMSRPR/IEC/2020/524) from the Institutional Ethics Committee at All India Institute of Medical Sciences (AIIMS), Raipur. The subjects of the study were patients diagnosed with intertrochanteric femur fractures (classified as Arbeitsgemeinschaft für Osteosynthesefragen (AO) Type 31 A1, AO Type 31 A2, AO Type 31 A3) who met the predefined inclusion criteria.

Inclusion Criteria

Intertrochanteric femur fractures (AO/Orthopaedic Trauma Association (OTA) Type 31 A1.1 to A3.3): No history of previous hip or lower limb surgery; patients willing to provide consent.

Exclusion Criteria

Pathological fractures: Previously non-ambulatory patients and patients with neurological disorders such as hemiplegia/paresis, paraplegia/paresis, quadriplegia/paresis; patients with associated fractures that might delay ambulation.

Intervention and data collection

Patients were clinically and radiologically evaluated to assess their suitability for surgery and to evaluate the fracture. Preoperative hematological and biochemical parameters, including serum vitamin D levels, were recorded. Patients who consented to participate were randomly assigned to one of two groups: Group A (fracture fixed using PFN) or Group B (fracture fixed using PFNA). So using a convenient sampling technique, a total of 50 patients (25 in each group) were enrolled within the defined study period. 

The surgeries were done under anesthesia and under an image intensifier. All operations were done on the fracture table. In all patients, the fracture was reduced by the standard technique of closed reduction. The reduction was confirmed with the help of an image intensifier with anteroposterior and lateral views.

In Group A, following reduction, entry was made just medial to the tip of the greater trochanter with awl under anteroposterior and mediolateral image intensifier guidance. A guide wire was passed. The femoral canal was opened with a manual proximal reamer. An appropriate-sized PFN was inserted over the guide wire. After proper positioning of the nail, a guide wire was inserted for the femoral neck screw and the derotation screw through the zig and their position was verified in the image intensifier. A drill hole was made for the derotation screw and an appropriate-sized screw was placed. Next, a drill hole was made for the femoral neck screw, and an appropriate-sized screw was inserted and compression was achieved. Distal locking was done through the zig in cases where short PFN was used and by free hand technique in cases with long PFN.

In Group B, following reduction, entry was made just medial to the tip of the greater trochanter with awl under anteroposterior and mediolateral image intensifier guidance. A guide wire was passed. The femoral canal was opened with a manual proximal reamer. An appropriate-sized PFNA was inserted over the guide wire. After proper positioning of the nail, the guide wire was inserted for the PFNA helical blade through the zig, and its position was verified in the image intensifier. A drill hole was made for PFNA helical blade and an appropriate-sized screw was hammered into place. Compression was achieved by locking the PFNA blade. Distal locking was done through the zig in cases where short PFNA was used and by free hand technique in cases with long PFNA.

**Figure 1:**
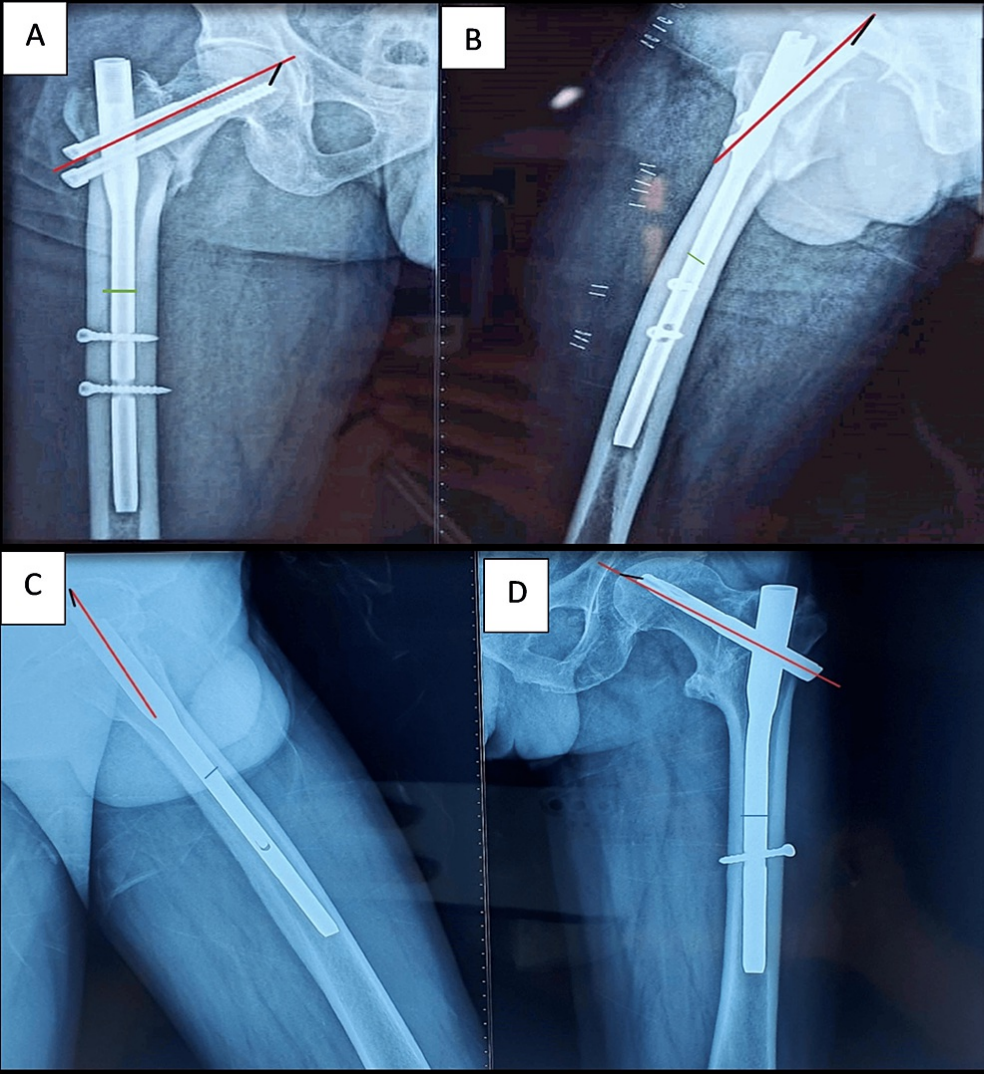
Tip-apex distance calculation using anteroposterior and lateral radiographs in (A, B) proximal femoral nail and (C, D) proximal femoral nail anti-rotation groups.

Radiological and clinical evaluations included anteroposterior radiographs of the pelvis with bilateral hips and lateral views of the affected hip with the thigh. The modified Harris hip score was used to assess functional outcomes, and parameters such as the tip-apex distance (TAD) and Cleveland index were measured to evaluate fixation quality. The TAD was calculated using the Baumgaertner formula (Figure 1) [7].

Intraoperative duration of surgery and blood loss were documented, and patients were followed for six months post-surgery, with evaluations at three and six months. Complications encountered during the follow-up period, such as deep infection, acetabular penetration, blade/screw migration, nail breakage, non-union, rotation failure, or screw/blade loosening, were recorded for each group.

Statistical analysis

Data collected were analyzed using the statistical software Statistical Package for Social Sciences (SPSS), version 22.0 (IBM Corp., Armonk, NY). Statistical tests were applied appropriately for comparison. Continuous variables were expressed as mean ± standard deviation (SD), and categorical variables as percentages. Two-sided p-values <0.05 were considered statistically significant. Descriptive statistics were performed for age, gender, and classification distribution. Chi-square tests were conducted to assess the association of union and complications with fixation type (PFN vs. PFNA) and to observe changes in the grading of modified Harris hip scores from three to six months in both groups. Independent t-tests were used to compare various parameters between the two groups, while paired t-tests were used to compare changes in mean modified Harris hip score over time within each group.

## Results

In our study, a total of 50 patients, 25 in each group were enrolled in the study. Both groups had 16 males and nine females. Thus, in both the groups and from a wider perspective, the study had 64.0% male participants and 36.0% female participants which shows that more males were presenting to the hospital with intertrochanteric fracture than females (p=1.000). The mean age of patients in the PFN group was 66.2 ± 9.8 years and that in the PFNA group was 66.4 ± 11.3 years which were comparable (p=0.936). The oldest patient operated on in this study was an 84-year-old female who underwent the surgery without any medical or surgical complications. In the PFN group, out of the total 25 patients enrolled, 13 had type 3A1 fractures (52.0%), 10 had type 3A2(40.0%) and two had type 3A3 (8.0%) fractures. The PFNA group had 13 patients (52.0%) type 3A1 fractures and nine patients (36.0%) had type 3A2 and three patients (12.0%) and 3A3 fractures (p=0.881). 

In our study, all the patients in both the PFN and PFNA groups showed fracture union at the six-month follow-up of the study, the radiographic evaluation showed the disappearance of the visible fracture line on both anteroposterior and lateral views and functionally were able to bear weight on the affected limb without pain (p=1.000). Both the groups studied showed no implant-related complications during the six-month follow-up period. Complications such as screw backout, implant cut-out, Z effect and reverse Z effect, implant breakage, and fracture in the femur shaft were not reported in any of the patients; both the implants performed equally well in this aspect of the study.

The PFNA group reporting a significantly lower mean blood loss compared to the PFN group in the study shows an intraoperative advantage of PFNA over PFN (p<0.001). The overall reduction in the duration of surgery in the PFNA group over the PFN group being statistically significant proves PFNA to be of advantage over the PFNA group (p<0.001) (Table 1).

**Table 1:** Comparison of blood loss and duration of surgery between the groups. PFN: Proximal femoral nail, PFNA: Proximal femoral nail anti-rotation.

Variables	Group PFN	Group PFNA	P value
	Mean ± SD		
Blood loss (ml)	123.6 ± 23.4	58.6 ± 18.6	<0.001
Duration of surgery (minutes)	98.2 ± 29.5	62.60 ± 15.9	<0.001

The value of TAD in both the groups is comparable without any significant difference therein, indicative of not much difference among the two groups when compared from the viewpoint of fracture fixation. TAD is also a predictor of implant-related complications with TAD > 25 mm considered to be an indicator of poor fixation, both the groups showing a mean TAD of 25 mm and along with that showing comparable implant-related complications further strengthen the correlation. However, a point worth consideration was that a total of six patients, three in each group had a TAD of > 25 mm (in the range of 25-30 mm each) but none of these patients showed any implant-related complications during the study duration of six months. The femoral neck-shaft angle is the measure of reduction following the fracture fixation, the angle averages between 125-130 degrees in the general population, and the Asian average measure has been reported at around 130 degrees in various studies, cut-off for coxa valga being > 140 degrees and coxa vara being <120 degrees. The average reduction was found to be satisfactory in both groups without any statistically significant difference (Table 2).

**Table 2:** Comparison of tip-apex distance and neck shaft angle between the groups. PFN: Proximal femoral nail, PFNA: Proximal femoral nail anti-rotation.

Variables	Group PFN	Group PFNA	P value
	Mean ± SD		
Tip-apex distance (mm)	21.8 ± 2.8	21.6 ± 2.9	0.826
Neck shaft angle (degrees)	130.8 ± 3.2	131.3 ± 3.4	0.555

**Figure 2:**
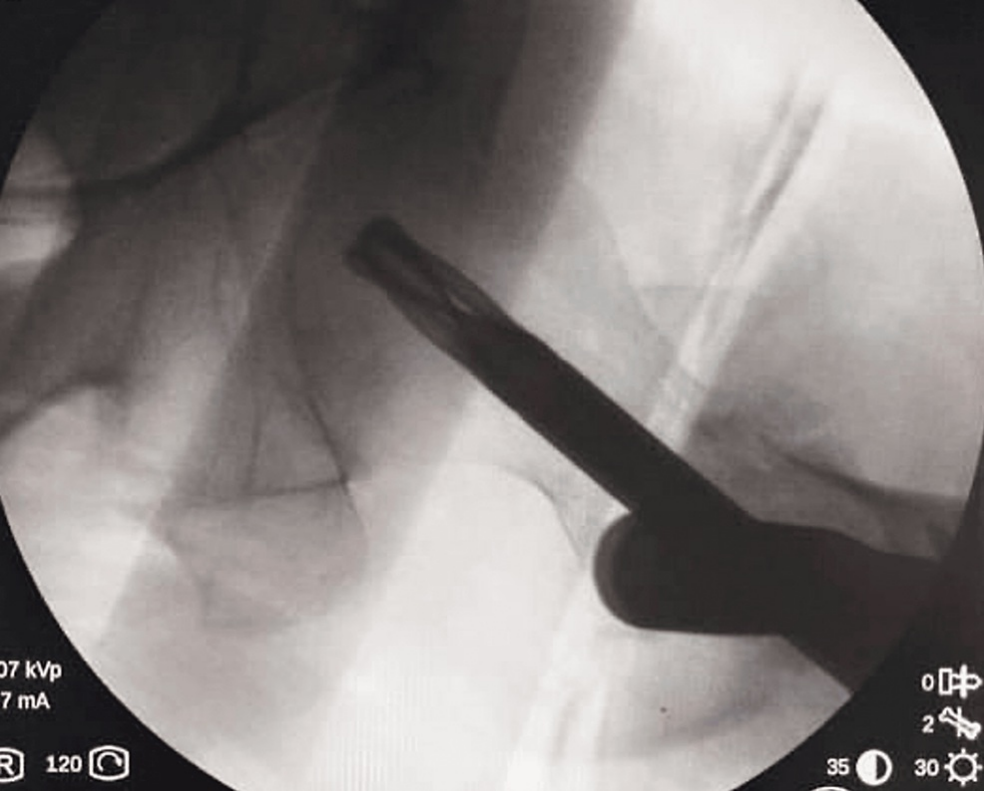
Center-center placement of helical blade (intraoperative) assessment.

The Cleveland index was center-center fixation (Figure 2) in more than half of the patients in both groups. Also, there was not any significant difference in the Cleveland index in both groups making radiological outcomes comparable (Table 3).

**Table 3:** Comparison of Cleveland index in both study groups. PFN: Proximal femoral nail, PFNA: Proximal femoral nail anti-rotation.

Cleveland index (placement)	PFN group	PFNA group	P value
Centre-centre	13(52.0%)	15(60.0%)	0.836
Centre-inferior	10(40.0%)	8(32%)	
Centre-superior	2 (8.0%)	2 (8.0%)	

The change in the modified Harris hip score in both groups is highly significant indicating improvement in the overall functional outcome of the patient at three months to that at six months. At the end of three months, 10 participants in the PFN group had a good functional outcome, fair outcome was seen in eight patients, and seven had a poor outcome, whereas, at the end of six months, eight patients had excellent outcomes in terms of the modified Harris hip score, four had good outcomes, 12 participants reported to have a fair outcome whereas only one participant reported to have poor outcome. In the PFN group, there was a rise from zero to eight in the excellent outcome group at the end of six months wherein poor outcomes decreased from a total of seven to just one. Also, in the PFNA group, there was a rise from zero to 11 in the excellent outcome group at the end of six months wherein poor outcomes decreased from a total of four to just zero. The change is highly significant statistically with a p-value < 0.001. Overall the modified Harris hip score was comparable in both groups at three months (p=0.152) and six months (p=0.119) with comparable outcomes (Table 4).

**Table 4:** Comparison of mean MHHS score/grade at three months and six months in both groups. PFN: Proximal femoral nail, PFNA: Proximal femoral nail anti-rotation, MMHS: Modified Harris hip score.

Groups	MMHS at 3 months	MMHS at 6 months	P value
	Frequency (%)/ Mean ± SD		
Group PFN	74.4 ± 9.0	84.9 ± 7.1	<0.001
Group PFN MMHS grade			
Excellent	0 (0.0%)	8 (32.0%)	<0.001
Good	10 (40.0%)	4 (16.0%)	
Fair	8 (32.0%)	12 (48.0%)	
Poor	7 (28.0%)	1 (4.0%)	
Group PFN	77.7 ± 7.2	87.7 ± 5.2	<0.001
Group PFNA MMHS grade			
Excellent	0 (0.0%)	11 (44.0%)	<0.001
Good	15 (60.0%)	13 (52.0%)	
Fair	6 (24.0%)	1 (4.0%)	
Poor	4 (16.0%)	0 (0.0%)	
MMHS score PFN	74.4 ± 9.0	84.9 ± 7.1	-
MMHS score PFNA	77.7 ± 7.2	87.7 ± 5.2	-
P value	0.158	0.118	-

Preoperative serum vitamin D levels were done in the early morning empty stomach samples of all the patients undergoing the surgery in both the study groups. Both the groups although comparable had marked deficiency of vitamin D at the time of sustaining the fracture, in the study 48 out of 50 patients that is 96.0% of the participants had serum vitamin D levels below 30 ng/ml when 30-100 ng/ml is considered to be the normal vitamin D levels in adults (Table 5).

**Table 5:** Comparison of mean serum vitamin D levels between the groups. PFN: Proximal femoral nail, PFNA: Proximal femoral nail anti-rotation.

Variables	Group PFN	Group PFNA	P value
	Frequency (%)/ Mean ± SD		
Vitamin D (ng/ml)	16.1 ± 6.7	16.5 ± 6.6	0.851
Vitamin D level			
< 20 ng/ml	19 (76.0%)	18 (72.0%)	0.943
20-30 ng/ml	5 (20.0%)	6 (24.0%)	
>30 ng/ml	1 (4.0%)	1 (4.0%)	

## Discussion

Intertrochanteric fractures are the most common type of hip fracture observed in the elderly, accounting for a significant portion of morbidity and mortality, constituting approximately 55.0% of all proximal femur fractures. As medical sciences advance, the global geriatric population is increasing, leading to a rise in hip fracture incidences, particularly intertrochanteric fractures. These fractures often result in high levels of disability, imposing social and economic burdens and predisposing patients with limited mobility to various medical complications [8-11]. Orthopedic surgeons frequently treat these fractures surgically, as surgery remains the primary treatment approach, especially for older patients. Surgical interventions aim to minimize intraoperative damage, thereby maintaining physiological balance and enabling early mobilization, which is crucial for avoiding postoperative complications and achieving optimal functional outcomes that enable independent daily living [12,13]. Over time, treatment modalities for these fractures have evolved, offering surgeons numerous implant options for internal fixation. However, the consensus on the optimal implant choice remains unclear. Currently, intramedullary nailing systems are widely regarded as the standard treatment modality for intertrochanteric fracture fixation, including unstable subtypes.

The average age of our study participants was 66.3 years overall, and both groups had a similar age distribution among the participants studied. In contrast, a study by Sharma et al. revealed a notable age difference between the PFN and PFNA groups, with the PFN group averaging 60.8 years and the PFNA group averaging 74.1 years [14]. However, our study showed no such disparity in age distribution between the groups [14]. Similarly, multicentric studies by Simmermacher et al., and Sadic et al., reported higher mean ages of 80.6 years and 73.6 years, respectively, in their study populations [15,16]. In addition, these findings implied that trochanteric fracture occurs early in the geriatric population, emphasizing the need for specialized medical and nursing care tailored to older patients. Additionally, surgical interventions should prioritize minimizing intraoperative damage to mitigate adverse outcomes, given the heightened vulnerability of this demographic to even minor disruptions in physiological equilibrium, which can significantly impact morbidity and mortality rates. In our study, out of the 25 patients enrolled in the two study groups, each group consisted of 16 males and nine females. Therefore, 64.0% of the total subjects studied were males, while only 36.0% were females. Sadic et al., conducted a study on trochanteric fractures and found a higher percentage of females compared to males, with a female-to-male ratio of 1.6:1, indicating a significantly higher number of females than males [16]. In contrast, Singh reported a slightly higher male predominance in their study, with 84 males and 68 females, resulting in a male-to-female ratio of 1.2:1 [17].

In our study, type 31A1 fractures were observed in 13 patients in both groups, making it the most common subtype overall, accounting for 52.0% in individual groups as well as the overall studied population. In a study by Singh, type 31A2 was the most common fracture type constituting 51.9% [17]. In our study, out of the total 50 patients enrolled in the study, all patients exhibited fracture union by the end of the six-month follow-up period, confirmed both radiologically and clinically. None of the treated fractures resulted in non-union, indicating the effectiveness of surgical treatment for these patients regarding fracture union. However, there was no statistical difference between the two implant groups in terms of fracture union, as indicated by a p-value of 1.00. Similarly, no implant-related complications were observed among the studied patients. In our study, up to the six-month follow-up period, no patient required re-operation due to implant-related complications. In a study by Simmermacher et al., they reported a union rate of 98.0% in trochanteric fractures treated using PFNA, with the majority (92.0%) achieving union within the first six months of follow-up [15]. Simmermacher et al., also noted that implant-related complications in 48 out of 313 total enrolled patients, resulting in an implant-related complication rate of 15.3%. However, a total of 117 patients experienced non-implant related complications, and 28 patients required re-operation [15].

In our study, the mean blood loss in the PFN group was 123.6 ml, while in the PFNA group, it averaged 58.6 ml, and this difference was found to be statistically significant. This establishes the clear superiority of PFNA over PFN in limiting intraoperative blood loss and, consequently, reducing surgical trauma for the patient. A similar study by Singh also found lower intraoperative blood loss in the PFNA group compared to the PFN group [17]. In a study by Park et al., the fixation of trochanteric fractures using PFNA resulted in a mean blood loss of 80.0 ml (ranging from 20 to 150 ml) [18]. In our study, in the PFN group, the mean total surgery duration was 98.2 minutes, while in the PFNA group, it was 62.6 minutes, and this difference was found to be statistically significant. Park et al., reported a mean intraoperative time duration of 82.5 minutes in patients undergoing fixation of proximal femoral fractures PFNA [18]. The simpler design of PFNA, with only one helical blade inserted in the proximal part and no requirement for reaming, saves considerable operative time. This reduction in intraoperative duration is particularly beneficial for the geriatric age group. Therefore, the superior design of PFNA over PFN can contribute to decreased anesthesia time and, consequently, reduced morbidity and mortality in patients with intertrochanteric fractures undergoing surgical fixation [8,12].

In our study, in the PFN group, the mean TAD was 21.8 mm, while in the PFNA group, it was 21.6 mm. Both groups showed comparable results in terms of TAD, indicating similar fracture fixation (p=0.826). A TAD of < 25 mm is considered acceptable in previous studies, indicating both implants performed equally well in radiological evaluation [19,20]. The neck shaft angle was measured and compared using immediate postoperative radiographs of the pelvis with bilateral hips. In the PFN group, the mean neck shaft angle was 130.8 degrees, while in the PFNA group, it was 131.3 degrees., the difference was not statistically significant. Singh, found similar results in their study, with mean neck-shaft angles of 130.6 degrees in the PFN group and 130.6 degrees in the PFNA group, showing no significant difference [17].

In our study, modified Harris hip scores at three and six months postoperatively were similar between both groups, with a significant increase in scores at six months compared to those at three months. Sharma et al., reported an average postoperative Harris hip score of 75.3 in the PFN group and 78.9 in the PFNA group, with a p-value of 0.54, indicating no significant difference between the groups [14]. Kashid et al. assessed the Harris hip score one year postoperatively and found a mean score of 86.8 in the PFN group and 88.5 in the PFNA group [21]. Similarly, Singh noted mean Harris hip scores of 79.1 in the PFN group and 79.3 in the PFNA group, with no significant statistical difference [17]. In our study, we did not report any complications. However, Sharma et al. observed fewer implant-related complications in patients treated with PFNA compared to PFN [14].

## Conclusions

In this prospective study, comparing proximal femoral nail (PFN) and proximal femoral nail anti-rotation (PFNA) for treating Intertrochanteric femur fractures, we found no statistically significant difference in radiological outcomes, including tip-apex distance, neck shaft angle, and Cleveland index. Both groups exhibited similar functional outcomes, with a good rating on the modified Harris hip score at three and six months postoperatively. Fracture union was achieved in all 50 patients across both groups by the end of the study period. However, PFNA showed significantly lower intraoperative time duration and blood loss compared to PFN, which is crucial, especially for the geriatric population prone to anesthesia-related complications. Overall, while both implants demonstrated comparable functional and radiological outcomes, PFNA exhibited advantages in reducing intraoperative complications, highlighting its potential benefits in clinical practice.
